# Effect of thermocycling on the flexural strength of porcelain laminate veneers

**DOI:** 10.4103/0972-0707.48835

**Published:** 2008

**Authors:** Deepa Subramanian, G Sivagami, D Sendhilnathan, CS Rajmohan

**Affiliations:** Department of Prosthodontics, Meenakshi Ammal Dental College and Hospital, Chennai, India

**Keywords:** Flexural strength, porcelain laminate veneers, thermocycling

## Abstract

**Aim:**

The aim of this study was to examine the impact of thermocycling on the flexural strength and development of surface flaws on the glazed surface of porcelain laminate veneer restorations with and without resin luting cement.

**Materials and Methods:**

80 Vitadur alpha dentin porcelain discs (10 mm diameter, 0.9 mm thickness) were glazed on one side and divided into two groups: A (porcelain laminate veneer only without resin luting cement) and B (porcelain laminate veneer luted with resin cement), each containing 40 discs. The discs in groups A and B were then thermocycled at different temperatures and were subjected to SEM analysis to evaluate the effect of thermocycling on crack propagation. Mean flexural strength was determined by using the ball-on-ring test. Student's * t* -test was used to find out the difference between strength values of the thermocycled porcelain discs and discs luted with resin cement.

**Results:**

SEM analysis revealed crack propagation in the subgroups subjected to extremes of temperature, i.e., 4 ± 1°C, 37 ± 1°C and 4 ± 1°C, 65 ± 1°C in the porcelain laminate veneers luted with resin cement. Flexural strength analysis revealed superior flexural strength for porcelain laminate veneers: 88.58 ± 6.94 MPa when compared to porcelain laminate veneers luted with resin cement: 8.42 ± 2.60 MPa. Results were tabulated and statistically analyzed using Student's * t* -test.

**Conclusion:**

Laminate veneer specimens exhibited greater flexural strength than those which were luted with resin cements. Laminate veneer specimens luted with resin cement and subjected to extremes of temperature, 4 ± 1°C and 37 ± 1°C and 4 ± 1°C and 65 ± 1°C, showed a marked decrease in flexural strength. After thermocycling at extremes of temperature, laminate veneer specimens luted with resin cement showed crack propagation. Fit of laminate veneers cannot / should not be compensated by the thickness of luting agent.

## INTRODUCTION

Dental ceramics were introduced nearly 150 years ago and have stood the test of time with their superior properties like biocompatibility, surface hardness, light absorption, light scattering behavior, and low electrical and thermal conductivity. In the course of their evolution, attempts were made to strengthen them to overcome their inherent brittle nature. Thus, the radical preparation executed in the making of crowns was eliminated to a greater extent by laminate preparations which could change the shape and color of teeth.[1] Ceramics function in the wet environment of the oral cavity and they deteriorate by slowly generating cracks, possibly due to the hydrolysis of silicate bonds.[2] These flaws are further aggravated by the stresses induced by thermal variations within the oral cavity.[3]

The clinical failure of porcelain laminate veneer restorations was due to the development of flaws on the glazed surface of the restorations. Postoperative cracking and failure of the restorations can occur as a consequence of thermal variations that these restorations are likely to encounter in service. Furthermore, the resin cement used for luting the laminate veneer may impose surface changes on the veneer when it is subjected to thermocycling.

In the above context, we had designed an *in vitro* study with the following objectives:

- To examine the impact of thermocycling on the development of surface flaws on the glazed surface of the restoration using scanning electron microscopy.
- To evaluate the biaxial flexural strength of porcelain laminate veneers with and without resin luting cement after being subjected to thermocycling.

## MATERIAL AND METHODS

### Fabrication of the test specimens

Eighty test specimens were fabricated in the form of discs of 10 mm diameter and 0.9 mm thickness using about 0.6 g of the Vitadur alpha dentine powder, preweighed in an electronic balance, and 0.22 mL of the modeling fluid measured with a micropipette.

The ceramic powder was mixed with modeling fluid, placed in the metallic mold, and compacted. The discs were then fired in a Multimat vacuum furnace (Mach 2 Dentsply), according to the manufacturer's instructions [Table 1].

**Table 1:** Firing cycle

Air-fired	600°C for 360 seconds
Vacuum-fired	970°C for 60 seconds
Air-fired	970°C for 60 seconds

The specimens were verified for dimensions using a micrometer. Heatless green carbide stones and emery discs were used to reduce the ceramic thickness. Specimens were divided into two groups of 40 specimens each, *i.e*., Group A: only *porcelain laminate veneer without resin luting cement*, and Group B: *laminate veneer luted with resin cement*. Group A test specimens were prepared as mentioned above whereas group B specimens had resin cement luted with the laminate veneers. First, discs of 0.9 mm thickness and 10 mm diameter were prepared. The unglazed surfaces of the discs were then etched with 5% hydrofluoric acid for 60 seconds. The etched surface was then washed thoroughly with water and luted to dual cure resin cement standardized to a thickness of 0.2 mm with the help of a metallic shim.

Test specimens from each of the two groups A and B were again divided into four subgroups according to the different temperatures employed for the thermocycling procedures. They are:

Subgroup (i) – 37 ± 1°C (control)

Subgroup (ii) – between 4 ± 1°C and 37 ± 1°C

Subgroup (iii) – between 37 ± 1°C and 65 ± 1°C

Subgroup (iv) – between 4 ± 1°C and 65 ± 1°C

### Thermocycling procedure

Thermocycling was done for both A and B groups. Each group had consisted of four subgroups of ten specimens each. The sample specimens of each subgroup were thermocycled as indicated above, between the maximum and minimum temperatures that the mouth is subjected to: 65 ± 1°C and 4 ± 1°C respectively, and the closed mouth temperature, 37 ± 1°C. Samples were carried in a mesh tray embedded in silicone putty impression material to expose only the glazed surfaces. The control group of samples belonging to subgroup (i) of groups A and B was submerged in a water bath maintained at 37 ± 1°C for the time equivalent to that required for 3500 cycles(equivalent to one year). The mesh trays with the samples from the other three subgroups of groups A and B were submerged in the respective water baths (corresponding to the indicating temperatures) for a constant length of time (five seconds).

The thermocycling unit was custom fabricated and consisted of a thermocouple and a heating element. A temperature sensor kept in the water bath was connected to a digital display unit. The digital display unit had a set button through which the temperature could be accurately set to ± 1°. When the water bath attains the desired temperature, the thermocouple automatically cuts off the power supply, thereby maintaining the set temperature. Temperatures of 37 ± 1°C and 65 ± 1°C were set with this unit.

A temperature of 4 ± 1°C was maintained with an ice pack containing crushed ice and the temperature was measured with a thermometer. The frequency of the thermocycling regime proposed in this study was based on the assumption that a maximum of ten extreme thermocycling cycles would occur per day. As a result, the 3500 cycles chosen would represent approximately one year of service for a porcelain laminate veneer restoration.

### Evaluation of surface topography using scanning electron microscopy

Each specimen in subgroups (i), (ii), (iii), and (iv) of both groups A and B were subjected to scanning electron microscopy analysis after they had been thermocycled. The specimens were prepared by platinum sputtering and analyzed using × 100 magnification.

### Evaluation of the flexural strength of laminate veneers

The ball-on-ring test was employed to assess the fracture strength of the surface-finished specimens. The test was performed using a Universal testing machine (*Instron*) with a loading ring apparatus at the displacement rate of 1.0 mm/min.

Breaking load values were obtained and the flexural strength of all the test specimens was calculated using the Timoshenko's equation:

$\mathrm{where}\sigma \max=\frac{P}{h^{\left[2\right]}}\left\{\left(1+v\right)\left[0.485\ln\left(\frac{a}{h}\right)+0.52\right]+0.48\right\}$

P = load at fracture in Newtons

h = thickness of the specimen (0.9 mm)

a = radius of the circle of support (5 mm)

n = Poisson's ratio of ceramic (0.25)

### Evaluation of flexural strength of laminate veneers luted with resin cement

Timoshenko's equation assumes a uniform elastic modulus and Poisson's ratio throughout the entire disc; hence, it could not be used in this form to calculate the biaxial flexural strength of the bilayered discs. First, the position of the neutral plate was calculated (h_ n_) and then, values for the elastic modulus (ε_*0*_) and Poisson's ratio (ν_*0*_) were obtained to represent the entire bilayered disc, *i.e.*, the ceramic luted with the resin cement. Finally, the biaxial flexural strength was assessed by evaluating the following equation:

${\mathrm{BFS}}_{\mathrm{bi}}=\frac{2\varepsilon_{1}\left(1-v_{0}\right)\mathrm{BFS}\left(T-h_{n}\right)}{\varepsilon_{0}\left(1-v_{1}\right)T}$

where

BFS is the flexural strength obtained

T = thickness of the disc (1.1 mm)

h_n_ = height of the neutral line from the top (0.55)

ε_1_ = elastic modulus of the resin cement (5 GPa)

ν_1_ = Poisson's ratio of resin cement (0.24)

ε_0_ = elastic modulus of the ceramic material (107 GPa)

ν_0_ = Poisson's ratio of ceramic material (0.25)

### Statistical analysis

Student's *t* -test was used to determine the difference between strength values of the thermocycled porcelain discs as well as discs luted with resin cement and that of the control groups.

## RESULTS

Eighty Vitadur alpha dentin discs were fabricated and divided into two groups: A consisting of only ceramic veneer discs and B consisting of ceramic veneer luted with resin cement. Each group was then divided into four subgroups based on the temperatures that they were subjected to during thermocycling. Breaking load values were obtained and flexural strength was calculated as shown in Tables 2 and 3.

**Table 2:** Flexural strength of group A specimens

Test samples(n)	Subgroup (i) Flexural strength (MPa)	Subgroup (ii) Flexural strength (MPa)	Subgroup (iii) Flexural strength (MPa)	Subgroup (iv) Flexural strength (MPa)
1	86.31	85.62	84.52	78.54
2	96.70	71.51	94.39	57.41
3	81.87	69.75	78.20	69.59
4	78.41	77.41	92.63	73.18
5	95.98	81.90	90.58	79.27
6	83.05	84.10	87.62	69.62
7	93.84	71.06	90.74	75.89
8	92.32	68.54	69.54	61.53
9	82.16	69.14	74.10	66.70
10	95.12	65.65	90.53	81.66
Mean	88.58	74.47	85.29	71.34
SD	6.94	7.9	8.51	7.91

SD–standard deviation

**Table 3:** Flexural strength of group B specimens

Test samples(n)	Subgroup (i) Flexural strength (MPa)	Subgroup (ii) Flexural strength (MPa)	Subgroup (iii) Flexural strength (MPa)	Subgroup (iv) Flexural strength (MPa)
1	09.80	5.61	09.08	7.01
2	11.64	4.99	11.67	5.44
3	10.35	5.45	10.03	5.43
4	11.04	7.01	10.46	4.99
5	11.52	5.38	11.54	6.78
6	11.67	6.77	10.95	6.63
7	10.83	7.04	10.71	6.83
8	11.43	5.78	11.25	5.51
9	10.19	4.82	11.43	5.91
10	10.47	5.52	11.49	6.35
Mean	10.89	05.84	10.86	06.09
SD	0.67	0.84	0.82	0.72

SD–standard deviation

Table 2 shows the flexural strength of group A samples wherein it was found that subgroup (i) had superior flexural strength followed by subgroups (iii), (ii), and (iv).

Table 3 shows the flexural strength of group B samples wherein it was again found that subgroup (i) had superior flexural strength followed by subgroups (iii), (iv), and (ii).

**Figure 1:**
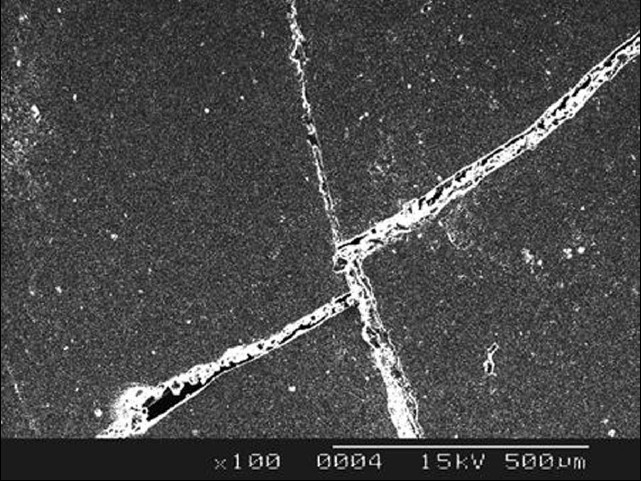
SEM picture of specimens belonging to group B subgroup (ii) specimens thermocycled between 4 ± 1°C and 37 ± 1°C

**Figure 2:**
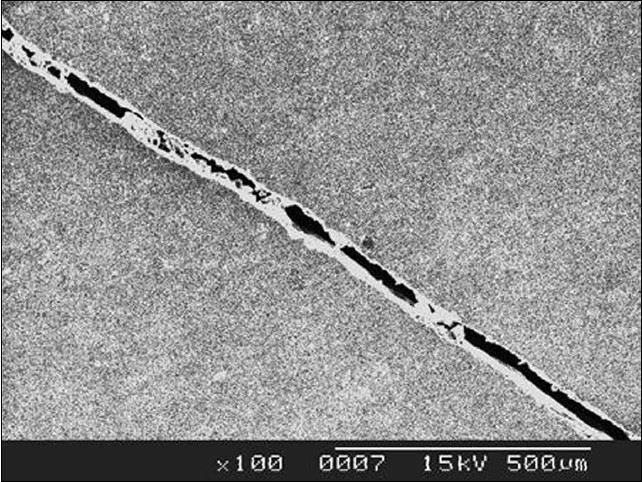
SEM picture of specimens belonging to Group B subgroup (iv) specimens thermocycled between 4 ± 1°C and 65 ± 1°C

SEM analysis was performed to evaluate the effect of thermocycling on crack propagation. Scanning electron microscopic analysis showed no significant findings in Group A specimens whereas the sub groups (ii) & (iv) of Group B specimens revealed cracks propagating through the ceramic veneer as shown in Figure 1 and Figure 2.

**Graph 1:**
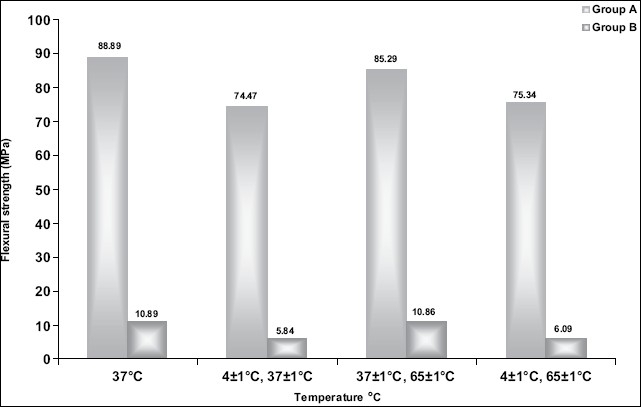
Comparison of flexural strength between Group A (Laminate veneer) and Group B (Laminate veneer luted with resin cement)

The results are tabulated and statistically analyzed using Student's *t* -test. The overall mean and mean values in all four subgroups' of flexural strength group A were significantly higher than the corresponding mean values in group B as shown in Graph 1 and Tables 4 and 5.

**Table 4:** Mean, standard deviation, and test of significance of mean values between groups A and B

Groups compared	Mean ± SD	*P* value*
Group A	79.92 ± 10.36	< 0.0001 (Sig.)
Group B	8.42 ± 2.60

* Student‘s independent *t*-test was used to calculate the *P* value

**Table 5:** Mean, standard deviation, and test of significance of mean values between groups A and B for each subgroup

subgroups compared	Group A	Group B	*P* value*
	Mean ± SD	Mean ± SD	
Subgroup (i)	88.58 ± 6.94	10.89 ± 0.67	< 0.0001 (Sig.)
Subgroup (ii)	74.47 ± 7.19	5.84 ± 0.81	< 0.0001 (Sig.)
Subgroup (iii)	85.29 ± 8.51	10.86 ± 0.82	< 0.0001 (Sig.)
Subgroup (iv)	71.34 ± 7.91	6.09 ± 0.72	< 0.0001 (Sig.)

* Student‘s independent *t*-test was used to calculate the *P* value

## DISCUSSION

Brittle materials such as ceramics fail because of the formation and growth of microscopic flaws that can form during the fabrication or service of these ceramics.[3–5] Ceramics are susceptible to slow crack growth at the tips of the surface flaws exposed to a moist environment as a result of hydrolysis of the silicate bonds.[1] Studies by White *et al*.[6] have shown that immersion of ceramics in water decreased their static strength and increased the crack velocity. Sherill and O'Brien,[4] Fairhurst *et al.,*[7] and Myers *et al*.[7] had demonstrated a decrease in flexural strength of aluminous and feldspathic porcelains when these were tested in water.

Furthermore, surface flaws may become extended due to thermal variations induced by ingested foods and drinks.[1] As attributed by Fleming *et al.*,[1] porcelain laminate veneers which are of only 0.5–0.9 mm thickness, may fail clinically due to the flaws extended as a result of thermal variations. Therefore, we sought to evaluate the effect of thermocycling on the flexural strength of the laminate veneers by themselves and when luted with resin cement.

Disc specimens were fabricated for the study to avoid the effect of flaws associated with rectangular bars.[8] The surfaces of the discs were glazed to increase the strength by inhibiting crack propagation through the compressive stresses generated on the surface of the ceramic during cooling.[9] Studies by Chu *et al*.[10] had proved that self-glazing was the most appropriate procedure to be carried out to control surface flaws in porcelain restorations if firing conditions were controlled properly.

Porosity affects crack propagation behavior of ceramics. Furthermore, Anusavice *et al*.[3] pointed out that irregular, nonspherical voids and not spherical voids facilitated crack initiation. Our study samples had spherical voids, and hence, it can be reasonably deducted that these voids have not affected the strength property of the material.

The thermocycling regimen was carried out between the maximal and minimal temperatures, *i.e*., 65 ± 1°C and 5 ± 1°C with the closed mouth temperature, 37 ± 1°C, being selected for the control subgroup. This is in accordance with the study by Palmer,[11] who showed that the maximal and minimal temperature extremes in an oral cavity ranged between 0°C and 65°C.

Three thousand five hundred cycles was the number of thermocycles chosen to approximate one year of clinical service for a porcelain laminate veneer restoration,[1] assuming that a maximum of ten extreme thermocycles would occur a day with a short dwell time of five seconds. After subjecting the samples to thermocycling, they were subjected to scanning electron microscopy which revealed crack propagation in the samples of group B, wherein ceramic discs were luted to resin cement and subjected to temperatures of 5 ± 1°C and 37 ± 1°C (subgroup (ii)) and 5 ± 1°C and 65 ± 1°C (subgroup (iv)).

As demonstrated by Magne *et al.*,[2] the occurrence of cracks was due to thermal variation that generated a cyclic mechanical load that resulted from the differential thermal expansion of the luting agent and the ceramic veneer. The co-efficients of thermal expansion of the luting agent and the porcelain veneer are 30°C×10^–6^ and 8°C×10^–6^ respectively.

Flexural strength of the specimens was measured because the strength of brittle materials is usually measured in flexure,[12] *i.e*., bending, because this test is generally easier to perform than a pure tensile test. The blunt-indentation technique suggested by White was used because unlike sharp indenters, blunt contact is favored in evaluating the evolution of damage.[6,13] Breaking load values were obtained for the ceramic discs of groups A (*laminate veneer*) and B (*laminate veneer luted with resin cement*) by subjecting them to tensile loading in a universal testing machine (*Instron*). The flexural strength values for group A were then calculated using Timoshenko's equation.[1] The flexural strength values for group B were evaluated using a formula for bilayered discs as proposed by Isgro *et al*.[14]

The flexural strength analysis revealed decreased strength of specimens of both groups A and B that had been subjected to extremes of temperature. This showed that lower temperatures had a deteoriating effect on the flexural strength of laminate veneers and laminate veneers luted with resin cement.

When group A specimens were subjected to extremes of temperatures, 4 ± 1°C and 37 ± 1°C and 4 ± 1°C and 65 ± 1°C, they showed decreased flexural strength due to detrimental tensile stresses at the veneer surface.[1]

Between the two groups, group B (*laminate veneers luted with resin cement*) specimens showed a marked decrease in flexural strength than those of group A (*laminate veneers only*). Among the specimens in group B, those that had been subjected to extremes of temperature, 4 ± 1°C and 37 ± 1°C and 4 ± 1°C and 65 ± 1°C, showed a decrease in their flexural strength.

This might be due to:

- Thermal variations which induced tensile stresses on the ceramic veneers.[2]
- Differences in the co-efficients of thermal expansion and the elastic modulus between the ceramic and the resin cement.[15]
- Water played the role of plasticizer, seeping into the resin cement and decreasing the elastic modulus of the resin.

## CONCLUSION

The conclusions drawn from the study are:

- Laminate veneer specimens exhibited greater flexural strength than those which were luted with resin cements.
- Laminate veneer specimens luted with resin cement and subjected to extremes of temperature, 4 ± 1°C and 37 ± 1°C and 4 ± 1°C and 65 ± 1°C, showed a marked decrease in flexural strength.
- After thermocycling at extremes of temperature, laminate veneer specimens luted with resin cement showed crack propagation.

The clinical implications are:

- Fit of laminate veneers cannot/should not be compensated by the thickness of luting agent. The resin cement used for luting porcelain laminate veneer actually decreases the flexural strength and causes crack propagation in the laminate veneer.
- During the laboratory phase of porcelain laminate veneer fabrication, the die spacer must be applied carefully to form a uniform layer. This is to avoid excessive thickness of the luting cement that would reduce the ceramic and luting cement ratio.
- Tooth reduction must be sufficient to ensure uniform ceramic thickness in the final restoration that would provide favorable ceramic and luting cement ratios.
